# Exploring Key Unmet Supportive Care Needs of Adolescent and Young Adult Cancer Patients: A Qualitative Study to Inform Regional Program Development

**DOI:** 10.3390/curroncol33070412

**Published:** 2026-07-10

**Authors:** Sitara Sharma, Sarah Cleyn, Haydn Bechthold, Alicia Hilderley, Amirrtha Srikanthan

**Affiliations:** 1 School of Human Kinetics, University of Ottawa, Ottawa, ON K1N 6N5, Canada; 2 Champlain Regional Cancer Program, The Ottawa Hospital Cancer Centre, Ottawa, ON K1H 8L6, Canada; 3 Patient Research Partner and Advisory Team, The Ottawa Hospital Cancer Centre, Ottawa, ON K1H 8L6, Canada; 4 Department of Medicine, University of Ottawa, Ottawa, ON K1H 8M5, Canada

**Keywords:** psychosocial oncology, fertility, mental health, sexual health, healthcare navigation, financial stress

## Abstract

**Background:** Adolescents and young adults (AYAs; aged 15–39) diagnosed with cancer face distinct challenges that are poorly addressed within traditional cancer care models. This qualitative study explored AYAs’ unmet supportive cancer care needs in Eastern Ontario (Canada) to inform the development of a tailored multidisciplinary program. **Methods:** As part of a larger mixed-methods study, AYAs receiving/post-cancer treatment in the Champlain region of Eastern Ontario were purposively recruited to complete a survey and a semi-structured interview. Demographic and interview data were analyzed descriptively and via thematic analysis, respectively. **Results:** Sixteen AYAs (M_age_ = 32.2 years [range: 19–42]; 56.3% female) were interviewed virtually using a co-designed, semi-structured guide between October 2024 and February 2025. Analysis revealed five themes (i.e., major care gaps) and 12 sub-themes, including: (1) *lack of standardized fertility counselling*, (2) *neglected psycho-emotional impact*, (3) *limited sexual health education and support*, (4) *difficulty navigating the healthcare system*, and (5) *financial toxicity and the cost of being sick young*. **Conclusions:** AYAs in Eastern Ontario face persistent gaps in supportive cancer care that undermine their quality of life. Our findings underscore the need for targeted system-level improvements and offer a foundation for co-designing an evidence-based regional AYA care model that better addresses the holistic needs of this growing population.

## Simple Summary

Young people diagnosed with cancer often face challenges that extend far beyond medical treatment, yet many feel unsupported in managing these experiences within current cancer care systems. Through interviews, 16 adolescents and young adults (aged 15–39) in Eastern Ontario (Canada) shared difficulties related to fertility, mental health, sexual health, healthcare navigation, and financial strain during and after cancer treatment. Many felt responsible for finding information, coordinating services, and advocating for themselves during an already overwhelming period of life. These experiences highlight important gaps in supportive cancer care for young people and show the need for more proactive, coordinated, and age-appropriate services. Results from this study will shape the development of a suitable regional program for adolescents and young adults affected by cancer and may also help guide future supportive care programs, policies, and research in other healthcare settings.

## 1. Introduction

Adolescents and young adults (AYAs), typically defined as individuals aged 15–39 years [1], represent a high-priority yet underserved population within oncology [2]. Each year, thousands of AYAs worldwide are diagnosed with cancer [3] during a critical life stage marked by transitions in education, employment, relationships, and identity formation. A cancer diagnosis during this period can disrupt career trajectories [4,5], delay or derail family planning [6,7], and result in long-term psycho-emotional [8] and financial strain [4,5,9], among other consequences. Nevertheless, continued reliance on cancer care models designed for pediatric or older adult populations fails to address the distinct biological and psychosocial needs of AYAs, thereby perpetuating disparities in survival outcomes known as the “AYA gap” [2].

Even within publicly funded healthcare systems such as Canada’s, where treatment costs and many specialist services are largely covered, AYAs face numerous unmet needs both during and after cancer care [10,11]. These needs are particularly high in sensitive but critical domains such as mental health, fertility, and sexual health [12], and noteworthy given existing initiatives aimed at improving timely access to psychosocial cancer services. Indeed, national organizations like the *Canadian Partnership Against Cancer* have developed frameworks outlining strategic priorities to enhance AYA cancer care, emphasizing the design and delivery of multidisciplinary, evidence-based, patient-centered services [13]. However, implementation of such guidelines has remained slow and inconsistent across the country [14]—a critical “knowledge-to-action” gap [15] that leaves many young people vulnerable within healthcare systems that are not built for them.

Policy efforts to strengthen AYA cancer care in Ontario (Canada) have gained momentum in recent years. In 2024, the *Pediatric Oncology Group of Ontario* and *Ontario Health* issued a joint call for regional proposals to enhance AYA services across the province; *The Ottawa Hospital (TOH) Champlain Regional Cancer Program* was among those selected to build a structured AYA program. As the sole tertiary cancer centre for the Champlain region in Eastern Ontario, TOH serves over 1.5 million people across 16 community hospitals, extending care to underserved areas like Baffin Island, Nunavut [16,17]. This region’s mixed geography (which spans urban, rural, and remote areas with varied access to care [18]) and cultural diversity (including strong presence of visible minority and Francophone communities [19]) underscore the need for a supportive care model that is tailored to reflect local realities. Despite TOH’s broad reach, no formal AYA pathway currently exists, and few (e.g., [20]) have sought regional data on AYA’s lived experiences with cancer, highlighting a critical opportunity for improvement.

Capturing and centering the voices of AYAs within specific healthcare settings is essential for identifying priority gaps and developing programs that are both relevant and implementable [19]. Accordingly, as a foundational step towards informing the co-design of a contextually grounded AYA supportive care model, the aim of this study was to explore AYAs’ lived experiences and unmet care needs within the Champlain region.

## 2. Materials and Methods

### 2.1. Study Design

This descriptive qualitative study was undertaken as part of a larger mixed-methods study investigating supportive care experiences and unmet needs among AYAs diagnosed with cancer in the Champlain region of Eastern Ontario (Canada). As this study was exploratory, it was not guided by a theoretical framework and no hypotheses were put forth; however, the existing literature and patient-engaged methods informed its development. Whilst the broader study also sought clinicians’ perspectives, patients’ experiences are the focus herein. To ensure transparency and completeness, reporting follows the *Consolidated Criteria for Reporting Qualitative Studies* (COREQ) checklist [21], as well as the *Guidance for Reporting Involvement of Patients and the Public* (GRIPP2) checklist [22] (see Supplementary Files S1 and S2, respectively).

### 2.2. Ethical Approval

This study was deemed by the *Ottawa Health Science Network Research Ethics Board* to fall within the scope of a “quality improvement project;” therefore, ethical approval was not required. Instead, this study was registered in the *IQ@TOH Project Registry* (registration date: 8 December 2024).

### 2.3. Participants and Procedure

Participants were recruited if they were: (1) aged 15–39 years at diagnosis, (2) currently receiving or had previously received treatment through TOH Cancer Centre, (3) diagnosed between 2015 and 2023, and (4) able to communicate in English (due to language constraints within the study team). Of note, although the AYA age range begins at 15 years [1], individuals younger than 18 were not included in this study because they received care at the *Children’s Hospital of Eastern Ontario* rather than TOH. Using TOH’s *MyChart* system (*Epic Systems Corporation*), patients were invited to complete an online survey, with the option to volunteer for a follow-up online interview. Of the 1315 patients contacted, 320 completed the survey, among whom 200 expressed interest in an interview. From this pool, 25 were purposively invited to aim for sample variation (e.g., in age, cancer type, treatment, parental status), and 16 individuals ultimately participated in interviews between October 2024 and February 2025.

### 2.4. Measures

#### 2.4.1. Survey

Participants completed a brief (~10 min) online survey housed on *Microsoft Forms* (affiliated with TOH) to gather information for sample description. This included items on: (1) sociodemographic characteristics (i.e., age, gender, ethnicity, religion, civil status, parental status, employment status), (2) medical history (i.e., age at diagnosis, cancer stage, type and treatments received or ongoing), and (3) care experiences and attitudes (i.e., key challenges encountered, perceived level of healthcare support, and aspects of care related to fertility preservation, sexual health, and mental health). Data pertaining to care experiences and attitudes are presented in a forthcoming manuscript and thus not reported herein.

#### 2.4.2. Interview

A semi-structured interview guide was co-designed with four AYA patient partners (including co-authors HB and AH, and acknowledged patient partner AL), focusing on cancer diagnoses, mental health, fertility, and sexual health (i.e., key areas of interest in AYA supportive care and survivorship [12]). Interviews were conducted virtually, audio-recorded using *Microsoft Teams* (affiliated with TOH), and lasted 46 to 60 min each. The interviewer (SC) was a female in her late 30s who has extensive clinical, administrative, and research experience with cancer patients (including young adults) through her career as a Registered Nurse and an Advanced Practice Nurse. She also received qualitative methods training from the corresponding author (AS) and through her graduate education. Together, her knowledge, skills, and experience equipped her to build rapport with participants and effectively explore their experiences within the healthcare system as AYAs navigating cancer.

Prior to conducting interviews, SC pilot-tested the interview guide with a young adult cancer survivor who was a patient partner on the broader study team and volunteered to take part (EA). This process was undertaken to assess the guide’s clarity, neutrality, logical flow, and feasibility for use within the allotted hour. This also allowed SC to practice probing and follow-up questioning. Data from this pilot interview are not presented, but feedback led to the removal of two redundant questions, the addition of one on financial toxicity, and re-ordering of items for improved flow; the full guide can be found in Supplementary File S3. Moreover, SC took field notes during interviews to help provide context for data analysis.

### 2.5. Sample Size

Given the exploratory nature of this study, no formal sample size calculation was performed. Rather, recruitment continued until data saturation was reached [23], with no substantially new themes identified after 16 interviews.

### 2.6. Data Analysis

Survey data were analyzed by the first author (SS) and AS using descriptive statistics. Interviews were automatically transcribed by *Microsoft Teams* and then verified for accuracy by an undergraduate research assistant (AP) and SC. AP conducted preliminarily coding (with input from SC and AS) and managed these data in *Microsoft Excel* (Version 16.105.3). Subsequently, SS, a psychosocial oncology PhD student trained in qualitative methods, reviewed the dataset in full and undertook an inductive thematic analysis [24] using *Microsoft Word* (Version 16.103.3). This involved six steps: (1) familiarization of the data, (2) systematic coding of meaningful content aligned with the study aim, (3) grouping related codes into sub-themes, (4) reviewing and organizing sub-themes into overarching themes, (5) refining and naming themes and sub-themes to reflect core meanings, and (6) selecting anonymized quotes to illustrate each theme and appropriately convey participants’ lived experiences. Themes and sub-themes were reviewed with AS, who provided feedback on the analytic structure and interpretation. In the interest of time and in light of emerging critiques [25], member checking was not conducted.

## 3. Results

### 3.1. Sample Description

Sixteen AYAs participated in this study; details can be found in Table 1. At baseline, they had an average age of 32.2 years (range: 19–42) and predominantly identified as female (*n* = 9; 56.3%), White (*n* = 11; 68.8%), and of Christian faith (*n* = 7; 46.7%). Most were partnered (*n* = 9; 56.3%), without children (*n* = 11; 68.8%), had completed post-secondary education (*n* = 11; 68.8%), and were employed (*n* = 13; 81.3%). With respect to medical characteristics, participants’ average age at diagnosis was 28.1 years (range: 14–37), and they most often reported having/undergoing stage III cancer (*n* = 6; 35.3%), Central Nervous System (CNS) or brain tumours (*n* = 7; 42.2%), and surgical treatment (*n* = 11; 36.7%).

### 3.2. Themes

**Figure 1:**
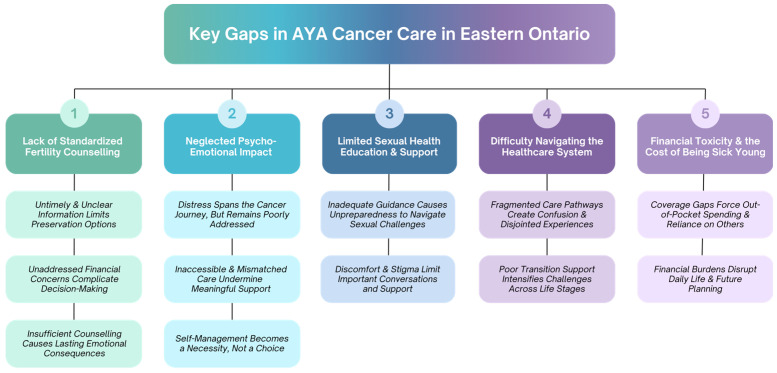
Identified gaps in cancer care for AYAs in Eastern Ontario.

As illustrated in Figure 1, five themes (i.e., key gaps in AYA care) comprising 12 sub-themes were developed from the data, including: (1) *lack of standardized fertility counselling*, (2) *neglected psycho-emotional impact*, (3) *limited sexual health education and support*, (4) *difficulty navigating the healthcare system*, and (5) *financial toxicity and the cost of being sick young*. Each (sub-)theme is detailed below and supported by quotations from pseudonym-identified participants; in quotations, […] indicates that text was omitted for clarity.

#### 3.2.1. Gap 1: Lack of Standardized Fertility Counselling

Participants consistently described gaps in fertility counselling, including untimely and unclear information and unaddressed financial concerns related to fertility, which led to lasting emotional consequences.

##### Untimely and Unclear Information Limits Preservation Options

For many, conversations about fertility after a cancer diagnosis never occurred, which some attributed to oncology healthcare providers seeming “averse to talking about fertility” (Quinn). When counselling *was* provided, it was often delayed. Indeed, several participants learned about fertility risks only *after* treatment had begun; that is, when they “[were not] in the right frame of mind” (Dana), or when “it was too late” (Tyler) for preservation opportunities. Some felt this timing of counselling positioned fertility preservation as a barrier to cancer treatment, forcing it to be deprioritized; Sandra noted, “any conversation around collecting [my] eggs would pause our ability to start radiation.” Moreover, when provided, fertility information was described to be “disorganized” (Georgia), “[in]explicit” (Quinn), and delivered through minimal resources like “one pamphlet or something like that” (Kyle).

##### Unaddressed Financial Concerns Complicate Decision-Making

Fertility counselling (or lack thereof) often failed to address the financial implications of fertility preservation. Although some participants were aware of relevant financial supports, many were uncertain about whether they could “justify that [kind of heavy] spending… especially when [they] need the funds for other things,” or were hesitant to pay “to store something that [they] don’t even know [they’re] going to use” (Daniel). Younger participants also expressed guilt about relying on loved ones for financial support; Tyler explained, “It was $500 a year to keep [my sperm] frozen… but I felt bad for making my parents pay that.” Participants suggested more flexible models, with Daniel proposing that AYAs should be able to “have [fertility material] stored, and if they do decide… [to] use it, *then* start enacting a cost.”

##### Insufficient Counselling Causes Lasting Emotional Consequences

While a few participants were indifferent or “just accepted it” (Jordan), many described regret and grief over lost opportunities to pursue biological parenthood. This was especially devastating for some without children; Sandra reflected that “having the choice ripped away is brutal… [it is] horrendous to go through.” Those who were parents also mourned the loss of future experiences. As Adele shared, “knowing… I’m not going to experience [having children] anymore… sometimes I get emotional about it.”

#### 3.2.2. Gap 2: Neglected Psycho-Emotional Impact

Psycho-emotional distress was a near-universal experience across the cancer care continuum, from the shock of diagnosis to ongoing anxieties in survivorship. Despite the pervasiveness of these concerns, psychological support was often inaccessible or poorly aligned with participants’ needs, requiring many to self-manage their emotional recovery.

##### Distress Spans the Cancer Journey, but Remains Poorly Addressed

Participants recalled profound emotional distress at diagnosis, including feeling “absolutely devastated” and “breaking down” (Gwen), as well as “wallowing [and thinking] why me?” (Georgia). Many also struggled with “coming to terms… [with] hav[ing] cancer” (Daniel), “fear of the unknown” (Adele), and being put “face to face with [their] own mortality” (Logan). Despite this turmoil, early psychosocial assessment or intervention was generally absent; as Jordan noted, “I was fine… but… no one has checked [in on] me… [or] assessed me.” During treatment, some accessed mental health care; however, structured support typically ended abruptly afterwards, leaving participants emotionally unprepared for the transition to survivorship. Simone explained, “you kind of stop receiving a lot of support…[but] survivorship is… the hardest part… I never felt like I really needed [mental health support] during treatment, but then afterwards…” Others found that distress persisted or resurfaced into survivorship, describing ongoing uncertainty, isolation, and fear of recurrence. For example, Daniel shared that his mental health has remained “very shaky,” and Quinn reflected that her “fear and anxiety kick up again” around significant cancer-related dates.

##### Inaccessible and Mismatched Care Undermine Meaningful Support

Psychological support was described as “the single most decisive thing in… recovering emotionally” (Sandra), yet meaningful care was often difficult to obtain. Cost was a major “barrier to entry” (Daniel); Simone emphasized that while psychotherapy was free during treatment, “that stops as soon as you’re out… it should be offered [for free] at least one year after[wards].” Beyond financial barriers, suitable care remained inaccessible. Gwen highlighted significant delays in accessing care, sharing that she is “currently waiting on community mental health… [but] it’s been two years,” while Daniel recounted multiple unsuccessful attempts to secure professional help, noting that “the first counselor completely blew [him] off, another one agreed there was a problem but fell through… a third said nothing [was] wrong with [him].” Short-term models of care further limited effectiveness; Kyle observed that “a lot of places offer four to six counseling sessions… [but] getting to know a therapist is two of those… [so] you’re [only] getting half the time.” Furthermore, several participants found available services to be “[not] super helpful” (Georgia) for their mental health needs or delivered by providers “so out of [their] depth dealing with cancer patients that [participants] just didn’t go back” (Logan).

##### Self-Management Becomes a Necessity, Not a Choice

With limited or inappropriate psychological support, participants often had to “manag[e] on [their] own” (Mason). Cited coping strategies included maintaining a sense of humour, staying active, enjoying hobbies and creative outlets, and spending time in nature (Simone, Gwen, Mason). Moreover, many sought emotional understanding and validation by surrounding or “distracting” (Daniel) themselves with friends, family, and partners. Peer networks offered additional emotional support; Simone noted, “The cancer community is super huge… it really helps [mentally] when you… find your tribe.”

#### 3.2.3. Gap 3: Limited Sexual Health Education and Support

Sexual health was recognized as important but was frequently overlooked. Inadequate guidance left participants unprepared to navigate sexual challenges, while discomfort and stigma—experienced by AYAs *and* clinicians—further limited conversations and support.

##### Inadequate Guidance Causes Unpreparedness to Navigate Sexual Challenges

Participants faced a wide range of sexual health and intimacy challenges but reported receiving little-to-no guidance or educational resources. When provided, information was often “limited… confusing… [and] unclear” (Quinn) or delivered via a “default” (Jordan) pamphlet or brochure, “but… you read those and… [learn] nothing” (Kyle). Although some received brief or partial guidance from clinicians, most, like Simone, did not “remember anyone talking to [them] about it or bringing anything up.” This left participants unprepared to navigate changes in sexual desire (e.g., “low libido” [Kyle]), physical comfort (e.g., painful sex [Gwen, Adele, Sandra]), or body image (e.g., “struggle[ing] to see [their] body[ies] as a thing [to] enjoy being in” [Georgia] due to scarring, hair loss, and feeling physically “broken” [Quinn]). Lack of guidance also created uncertainty around what was safe during or after treatment; for example, some feared that engaging in sexual activity while their bodies contained treatment-related toxins would cause “adverse health effects for [their partners]” (Logan). Simone explained, “It would have been nice to have some more information on how I could make [navigating these challenges] easier,” and Logan emphasized the importance of “offer[ing] [information] up front before it becomes an issue, especially when you’re dealing with young couples.”

##### Discomfort and Stigma Limit Important Conversations and Support

Concerns around sexual health often went unspoken due to discomfort and stigma. Participants described it as a “taboo topic” (Daniel) that they were “too embarrassed to approach on [their] own” (Logan), particularly at younger ages; Daniel reflected, “Now, I don’t have a problem with it… [but] at 18… I would have been a bit more uncomfortable and awkward.” Such discomfort was not limited to participants; clinicians were also perceived to be uneasy. Georgia noted that some nurses appeared comfortable discussing sexual health, though her oncologists were “pretty squirmy,” suggesting that they might “[feel] awkward, or maybe they don’t see [sexual health education] as part of their [professional] role.” Some participants also felt clinicians made unrealistic or even “insensitive” (Sandra) assumptions about their sexual health, reinforcing silence around the topic. When conversations *did* occur, they were often with providers *outside* the core cancer team, such as gynecologists, who were perceived to be more “comfortable discussing these issues” (Georgia). Sandra highlighted that it “would have been helpful” if trained clinicians had proactively initiated such conversations.

#### 3.2.4. Gap 4: Difficulty Navigating the Healthcare System

Fragmented care pathways and insufficient support during major life transitions made navigating the healthcare system a significant challenge for participants.

##### Fragmented Care Pathways Create Confusion and Disjointed Experiences

Participants described navigating a healthcare system that felt disconnected, with care quality varying across teams and settings. For instance, Logan “had an amazing oncology team… but [his] surgical team… was just not good.” Many were bounced between healthcare providers or sites, leading to contradictory guidance and inefficiencies. Jordan recalled that he had “spoken to maybe six different doctors… triage and so on… they sen[t] [him] to the nearest hospital… then they sent [him] back.” Fragmentation often resulted in repeated assessments and “having to redo the [same] conversation over and over again… [to individuals] not part of the existing care team,” which Georgia deemed “exhausting.” Participants also struggled to identify *who* was responsible for addressing their concerns; Giulia “got most of [her] answers from a student in the plastic surgery department… [who was] probably not the [right] person,” while Georgia observed clinics were “focused on a very narrow set of symptoms… [and] no one addressed broader issues.” Limited follow-up and support compounded confusion; Quinn described significant “information overload” and emphasized that “repeating information or following up with an e-mail” would support greater understanding and retention of guidance.

##### Poor Transition Support Intensifies Challenges Across Life Stages

Participants highlighted major life transitions (e.g., moving from pediatric to adult care or shifting from treatment to survivorship) as points where support dropped off. Tyler, for example, had access to a therapist at the local children’s hospital “before ag[ing] out,” and Josephine reported losing connection to child life services once she entered adulthood. Kyle shared that “adapting… back to regular life” was also difficult because “the different requirements of life are more challenging… [and] unfortunately… [he is] disabled now.” Similarly, Steven reflected that “as a young person, it’s… normal to… go work hard and… leave your body on the side because you feel invincible,” but this sense of invincibility changes post-cancer and can be hard to adjust to. Further, treatment intensity left many with little capacity to plan for future milestones; Simone explained that when “you’re diagnosed and going through treatment… you’re not really thinking about anything else but… surviving… that’s when a lot of [other] people start to settle down in their life and it’s like, holy… everything is different now.” To help ease transitions, participants emphasized the value of timely, age-appropriate resources. As Quinn put it, “Are there organisations… [or] anyone that I can talk to that’s closer to my age with cancer?… Getting some of those resources up front at a more opportune time… or repeated again… could probably be helpful.”

#### 3.2.5. Gap 5: Financial Toxicity and the Cost of Being Sick Young

High costs of treatment, limited coverage, and life-stage vulnerabilities combined to create significant out-of-pocket costs, financial reliance on others, and disruptions to both daily life and future planning.

##### Coverage Gaps Force Out-of-Pocket Spending and Reliance on Others

Participants reported substantial costs for medications, treatments, and supportive care that were often only partially covered. Kyle explained, “The chemo drugs weren’t covered because they were out-of-hospital pills, so that cost an arm and a leg… My mom’s health insurance paid for a lot of it, and we paid the rest.” Likewise, Georgia noted, “I was fortunate to have long-term disability coverage… but it’s only 65% of your income.” Some supportive therapies, like cannabis, were entirely uncovered; Quinn said, “I know… there’s a lot of policy reasons for that, [but] it was the only thing that would help me get to sleep… [and since] we had no coverage for that, it was all… [paid] out of pocket.” For many, especially younger participants, these shortfalls created significant financial strain and forced reliance on loved ones or community support. Daniel reflected, “At 18… the amount of money that you do have is limited… My grandpa… was making sure I had financial support,” while Tyler and Josephine described setting up GoFundMe pages to help raise funds for medical expenses.

##### Financial Burdens Disrupt Daily Life and Future Planning

High costs, lost income, and ongoing expenses created long-term financial strain that affected participants’ abilities to save money, work, or plan for their futures. Simone remarked, “My partner doesn’t live here anymore… I’m living paycheck to paycheck… I have $0 that I’m able to save… my credit has gone to complete trash,” and Sandra observed “los[ing] out on some income [while] being on long-term disability,” adding, “[my partner and I are] fine… [but] I can see how this financially ruins people.” Participants also highlighted age-specific vulnerabilities; Sandra explained, “You’re at a stage as a young adult… where you’re building your career… you’re just starting to try and put some [money] away… [or] paying off school.” Ancillary costs (e.g., travel and parking for treatment) further compounded financial burden. Georgia shared, “[My oncology centre] is three and a half hours away… travel costs really add up… Even though you can claim it back in taxes, being away from home, parking, eating your meals out… it adds up.” Accordingly, a need for targeted support was emphasized, with Gwen voicing that “especially for younger people who are working, having resources and support for financial burdens… is very important… In this day and age, it’s so hard to get a job.”

## 4. Discussion

The purpose of this study was to explore the unmet needs of AYA cancer patients who were receiving or had previously received treatment in the Champlain region of Eastern Ontario (Canada) as a foundation for developing a tailored supportive care model. Findings from interviews with 16 AYAs highlight important gaps in fertility, mental health, sexual health, healthcare navigation, and financial support, which are issues that are well documented in the AYA oncology literature [26,27,28,29]. The persistence of these gaps despite care delivery within a publicly funded healthcare system suggests that universal coverage alone may be insufficient to ensure access to comprehensive, developmentally appropriate care. Rather, these findings point to broader structural limitations in cancer care models that are not designed to meet the unique life-stage needs of AYAs and highlight ongoing challenges in translating established national AYA priorities [13,30] into real-world care. By providing in-depth, context-specific insight into patients’ supportive care experiences within the Champlain region, this study extends prior work beyond identifying unmet needs to illustrating *how* and *why* they persist in practice, while offering clinical and research implications for regionally tailored solutions.

A key insight from this study is that regional AYA cancer care appears largely *reactive* as opposed to *proactive*, with critical misalignment between the timing of need and the timing of care across the cancer continuum. Across domains, support was delayed (e.g., fertility counselling was not introduced until preservation options were limited), time-bound (e.g., mental health services were abruptly withdrawn or became difficult to access post-treatment despite ongoing distress), or absent altogether (e.g., sexual health concerns were seldom addressed unless raised by participants). These patterns suggest AYA supportive care is often initiated in response to emerging concerns or patient self-advocacy, rather than being systematically embedded at key clinical milestones in anticipation of their unsurprising needs. Consequently, opportunities for early (i.e., as close to cancer diagnosis as possible) psychosocial screening or intervention may be missed, despite evidence that such timely support can act as a “gateway” to better long-term outcomes (e.g., functional status [31], quality of life [32]). This timing mismatch was particularly concerning for participants during survivorship, a period in which they felt ongoing or intensifying needs (e.g., emotional distress) alongside a stark reduction in available support (e.g., mental health services). Similar “cliff effects” have been reported across diverse international healthcare settings, including the United States, the United Kingdom, and France [33,34,35]; this suggests existing AYA care models remain front-loaded despite persistent post-treatment needs, regardless of differences in healthcare system structure. Accordingly, rather than representing an endpoint, AYA survivorship may require reconceptualization as an active period of care comprising proactive, structured, and longitudinal support (e.g., tailored survivorship care plans and coordinated follow-up pathways [36,37,38]), alongside more gradual tapering of services (e.g., extended access to psychosocial supports [37]).

Aligned with the AYA literature [12,39], findings also highlight gaps in regional AYA care stemming from silence around “taboo” yet developmentally salient topics like fertility and sexual health, which participants described as rarely addressed by clinicians. This suggests these domains remain structurally deprioritized within cancer care in spite of established (inter)personal consequences following cancer [40] and clinical practice guidelines emphasizing their importance (e.g., [41,42]). These findings are particularly notable in Ontario, where fertility specialist consultations and fertility preservation for cancer-related infertility are publicly funded and oncology referrals are typically expedited because of the time-sensitive nature of treatment [43]. The persistence of these gaps despite such supports indicates that access may be limited by inconsistent implementation of supportive care communication and referral pathways. Indeed, prior research has identified barriers to appropriate fertility and sexual health education and intervention, including clinician-level factors (e.g., discomfort, uncertainty regarding role responsibility, resource constraints, competing clinical priorities, and limited knowledge, training, or capacity [14,44]) and patient- and caregiver-level factors (e.g., embarrassment, stigma, and perceived discomfort from clinicians [45]). Taken together, it seems silence around fertility and sexual health is co-produced, reinforcing mutual avoidance and leaving AYAs without guidance on issues central to identity, relationships, and future planning. In the case of fertility, this has clear downstream consequences, with participants describing high-stakes, often uninformed decisions made under time pressure, emotional distress, and financial uncertainty due to inadequate fertility discussions. Addressing this crucial gap requires embedding such discussions as routine, normalized components of AYA care. Possible strategies include: (1) integrating proactive, standardized fertility counselling, (2) ensuring clinicians are well-trained on these topics to be able to adequately provide education, address concerns, and facilitate a safe space for discussion, and (3) providing routine assessment of fertility and sexual health needs into AYA care pathways. Published AYA-specific communication and assessment guidance (e.g., sample probes, talking points, key tools) for such needs [12,46,47] can provide clinicians with a practical starting point.

Findings further suggest that financial toxicity, another often under-discussed or “taboo” topic in AYA oncology [12], is insufficiently addressed by clinicians in this regional setting. Similar to fertility and sexual health concerns, financial issues were rarely raised proactively with participants, despite their substantial influence on care experiences and decision-making. This left AYAs to manage financial strain on their own, often relying on informal supports (e.g., personal savings, family and friends), as observed in prior research conducted across both publicly and privately funded healthcare systems [48,49]. Although income replacement lies outside the scope of the Canadian healthcare system, other system-level responses are critically needed to improve healthcare affordability and reduce financial burden among AYAs, a population especially vulnerable to economic disruption [9]. These may include integrating early financial screening and standardized financial counselling or navigation [37,50,51] into AYA care programs to identify at-risk patients and better support them in managing financial hardship associated with cancer.

Furthermore, challenges are compounded by fragmentation across the cancer care continuum in regional AYA cancer care. Akin to previous research [33,49,52], participants described disjointed care pathways, inconsistent communication with/between clinicians, and uncertainty regarding *who* was responsible for addressing their supportive care needs, particularly during transitional periods (e.g., pediatric-to-adult care and treatment-to-survivorship). In the absence of coordinated support, many independently sought mental health services, navigated complex systems, and sourced information related to fertility, sexual health, and financial concerns. While such behaviours are often framed as “self-management” (i.e., active patient involvement in one’s own care [53]), our findings suggest they may instead reflect gaps in system-level support and a transfer of responsibility from healthcare systems to patients. This distinction is important, as self-management is conceptualized as empowerment within a supportive context [54], not a substitute for absent services. Expecting AYAs to coordinate their own care, particularly for sensitive or stigmatized concerns as discussed above, may therefore add burden to individuals already navigating complex medical and psychosocial challenges. Notably, reliance on patient-driven navigation raises important equity considerations as those with greater financial, social, or informational resources may be better positioned to access or engage with support, potentially widening disparities in care [55,56]. Collectively, these findings highlight the need for integrated, anticipatory, and longitudinal care models that reduce reliance on self-advocacy and better support young people across the cancer continuum, including during key transitional periods. Helpful approaches may include integrating AYA care navigators into healthcare pathways to ease transitions and ensure timely follow-up and continuity of care [57,58,59], as well as offering complementary psychosocial and family supports (e.g., AYA-specific peer support, caregiver education and training) to address ongoing supportive care needs.

### 4.1. Clinical and Research Implications

Several opportunities for *system-level* (i.e., clinical) improvement emerge from the data, organized by theme. First, there is an urgent need to standardize fertility counselling at diagnosis, including clear timelines, options, and costs seeing as delays and inconsistencies contributed to lasting distress among many participants. Second, long-term mental health support should be embedded into AYA survivorship care plans, given the enduring impact of anxiety and fear of recurrence post-treatment. Third, sexual health conversations must be normalized and comprehensively integrated into oncology care; providers require training and tools to initiate open, inclusive conversations about intimacy, sexual identity, and relationships. Fourth, AYA care navigators represent a promising system-level approach to reducing care fragmentation during and after treatment. Last, efforts to mitigate financial toxicity are critical; oncology teams should proactively offer financial counselling, especially for AYAs navigating early career or educational stages.

Our findings also offer key *research* implications. First, future AYA initiatives should continue to be co-designed with young people using participatory and community-based research methods to ensure supports reflect their lived experiences and needs. Second, longitudinal research is needed to examine how concerns identified herein (e.g., fertility regret, fear of recurrence, post-traumatic stress, financial strain) may evolve over time and to inform the development of sustainable, long-term support systems. Third, further exploration of clinician perspectives (e.g., [60]) is critical to better understand systemic barriers, facilitators, and resource needs associated with delivering developmentally appropriate AYA cancer care. Finally, developing and evaluating interventions targeting key unmet needs (e.g., standardized fertility pathways, psychosocial screening approaches, care navigation models, financial support initiatives) may help identify effective strategies to improve supportive care, especially for underserved populations.

Importantly, implementing these recommendations will require addressing broader structural challenges within existing cancer care systems. A key challenge is that current oncology models at adult centers are largely designed around older adult populations and may not adequately accommodate the developmental and psychosocial needs of AYAs [13]. The variability in supportive care described by participants suggests that, in the absence of standardized AYA care pathways, the identification and coordination of supportive care may rely heavily on individual clinicians, potentially contributing to inequities in access and continuity of care across settings. Furthermore, oncology workforce shortages, burnout and well-being concerns, competing clinical priorities, and limited psychosocial resources may impede delivery of comprehensive supportive care [61,62,63]. Moving toward more proactive AYA care may therefore require strengthening multidisciplinary collaboration, expanding the roles of non-physician providers (e.g., nurses, social workers, pharmacists) within oncology settings, leveraging community resources, and establishing clear pathways for screening, referral, and follow-up across the cancer continuum [13,64].

### 4.2. Limitations and Strengths

Our results should be considered in light of various limitations: (1) this study focused on a single region which can limit generalizability; (2) adolescents (i.e., those aged 15–17) were not able to be recruited herein; thus, findings may not adequately capture the perspectives of younger AYAs who may experience cancer differently due to their life stage; (3) the perspectives of LGBTQ2+ and racialized youth were not adequately captured in this sample, which are particularly important when discussing sexual health and care equity (e.g., [29]); (4) individuals with breast cancer were not represented, despite it being the most common solid cancer type in AYAs [3]; (5) due to language constraints within the study team, the study was conducted in English, which may have excluded Francophone individuals who wished to voice their experiences in a region with a considerable Francophone population; (6) the findings largely reflect the voices of AYAs diagnosed with stage III or IV disease, whose treatment experiences and survivorship concerns may differ from those of individuals with earlier-stage cancers; (7) participants were predominantly White and educated, further limiting representativeness; and (8) self-selection bias may have led those with stronger or more negative experiences to participate.

Even so, this study had strengths, including the following strategies that were implemented to enhance study rigour and trustworthiness: (1) the interview guide was co-designed and pilot tested with young adult cancer survivors (including HB, AH, and AL) to ensure questions were relevant; (2) open-ended questions centered participant voices, allowing them to express what they felt was important and expand upon or alter responses as they wished; (3) the interviewer built rapport through empathetic engagement, supporting a constructive dialogue; (4) data were analyzed systematically and reflectively by SS and AS, who considered multiple interpretations of the data while acknowledging their preconceptions, personal experiences, and prior knowledge; and (5) reporting followed established checklists for reporting qualitative research (i.e., COREQ [21]) and patient engagement (i.e., GRIPP2 [22]).

## 5. Conclusions

In conclusion, AYA cancer patients in the Champlain region face systemic gaps related to fertility counselling, mental health support, sexual health guidance, healthcare navigation, and financial care. These gaps are not just clinical; they also affect quality of life, relationships, and long-term outcomes. Insights will directly inform the co-design of a regional care model to meet AYAs’ needs and improve supportive cancer care in Eastern Ontario, while also offering system-level and research opportunities for other regions to consider.

## Figures and Tables

**Table 1:** Baseline sociodemographic and medical characteristics (*n* = 16).

Variables	Values
**Sociodemographic Characteristics**	
Current Age (M years ± SD; range)	32.2 ± 6.7; 19–42
Gender, *n* (% female)	9 (56.3)
Ethnicity, *n* (% White)	11 (68.8)
Religion, *n* (% Christian) ^a^	7 (46.7)
Civil Status, *n* (% partnered)	9 (56.3)
Parental Status, *n* (% not parents)	11 (68.8)
Highest Level of Completed Education, *n* (% post-secondary)	11 (68.8)
Vocational Status, *n* (% employed)	13 (81.3)
**Medical Characteristics**	
Age at Diagnosis (M years ± SD; range)	28.1 ± 7.5; 14–37
Cancer Stage, *n* (%) ^b^	
0 (in situ)	1 (5.9)
I	3 (17.6)
II	3 (17.6)
III	6 (35.3)
IV (metastatic)	4 (23.5)
Cancer Type, *n* (%) ^b^	
Central Nervous System/Brain	7 (41.2)
Hematological	4 (23.5)
Gynecological	3 (17.6)
Gastrointestinal	2 (11.8)
Melanoma	1 (5.9)
Cancer Treatment, *n* (%) ^c^	
Radiation	7 (23.3)
Surgery	11 (36.7)
Systemic Therapy	12 (40)

*Notes.* SD = standard deviation. ^a^ *n* = 15 participants. ^b^ One participant reported two cancer stages/types at baseline; thus, the denominator here is 17. ^c^ Several participants received/were receiving more than one cancer treatment; thus, the denominator here is 30.

## Data Availability

The data presented in this study are available on request from the corresponding author due to privacy reasons.

## Supplementary Materials

The following supporting information can be downloaded at: https://www.mdpi.com/article/10.3390/curroncol33070412/s1, File S1: *Consolidated Criteria for Reporting Qualitative Research* (COREQ) Checklist; File S2: *Guidance for Reporting Involvement of Patients and the Public* (GRIPP2) Checklist; File S3: Interview Guide.

## Abbreviations

The following abbreviations are used in this manuscript:

AYA	Adolescent and young adult
COREQ	Consolidated Criteria for Reporting Qualitative Studies
GRIPP2	Guidance for Reporting Involvement of Patients and the Public
TOH	The Ottawa Hospital

## References

[1] National Cancer Institute Adolescents and Young Adults with Cancer. https://www.cancer.gov/types/aya.

[2] Adolescent and Young Adult Oncology Progress Review Group (2006). Closing the Gap: Research and Care Imperatives for Adolescents and Young Adults with Cancer.

[3] Li W., Liang H., Wang W., Liu J., Liu X., Lao S., Liang W., He J. (2024). Global cancer statistics for adolescents and young adults: Population based study. J. Hematol. Oncol..

[4] Altherr A., Bolliger C., Kaufmann M., Dyntar D., Scheinemann K., Michel G., Mader L., Roser K. (2023). Education, employment, and financial outcomes in adolescent and young adult cancer survivors-a systematic review. Curr. Oncol..

[5] Janssen S.H.M., van der Meer D.J., van Eenbergen M., Manten-Horst E., van der Graaf W.T.A., Husson O. (2022). Short- and long-term impact of cancer on employment and financial outcomes of adolescents and young adults (AYAs): A large population-based case-control registry study in the Netherlands. ESMO Open.

[6] Benedict C., Stal J., Davis A., Zeidman A., Pons D., Schapira L., Diefenbach M., Ford J.S. (2023). Greater fertility distress and avoidance relate to poorer decision making about family building after cancer among adolescent and young adult female survivors. Psycho-Oncology.

[7] Jakel K., Richter D., Leuteritz K., Sender A., Hinz A. (2023). Sexuality, fertility, family planning, family life, and partnership in young breast cancer patients: A longitudinal study. Front. Psychol..

[8] Itzep N., Roth M. (2022). Psychosocial distress due to interference of normal developmental milestones in AYAs with cancer. Children.

[9] Thom B., Friedman D.N., Aviki E.M., Benedict C., Watson S.E., Zeitler M.S., Chino F. (2023). The long-term financial experiences of adolescent and young adult cancer survivors. J. Cancer Surviv..

[10] Jones J.M., Fitch M., Bongard J., Maganti M., Gupta A., D’Agostino N., Korenblum C. (2020). The needs and experiences of post-treatment adolescent and young adult cancer survivors. J. Clin. Med..

[11] Guirguis S., Fitch M., Maganti M., Gupta A.A., D’Agostino N., Korenblum C., Jones J.M. (2021). Biopsychosocial factors associated with supportive care needs in Canadian adolescent and young adult cancer survivors. J. Clin. Med..

[12] Perez G.K., Salsman J.M., Fladeboe K., Kirchhoff A.C., Park E.R., Rosenberg A.R. (2020). Taboo topics in adolescent and young adult oncology: Strategies for managing challenging but important conversations central to adolescent and young adult cancer survivorship. Am. Soc. Clin. Oncol. Educ. Book.

[13] Canadian Partnership Against Cancer (2019). Canadian Framework for the Care and Support of Adolescents and Young Adults with Cancer.

[14] Rutkowski N., Beattie S., Schulte F., Thurston C., Boychuk A., de Guzman Wilding M., Korenblum C., Tutelman P.R. (2026). An environmental scan of services for adolescents and young adults diagnosed with cancer across Canadian pediatric and adult tertiary care centres. Curr. Oncol..

[15] Graham I.D., Logan J., Harrison M.B., Straus S.E., Tetroe J., Caswell W., Robinson N. (2006). Lost in knowledge translation: Time for a map?. JCEHP.

[16] Roberts C. (2024). Breaking barriers: Indigenous nurse navigator role in oncology care for the Inuit. Can. Oncol. Nurs. J..

[17] The Champlain Regional Cancer Program (2025). Regional Cancer Plan 2024–2028. https://cdn.phototourl.com/pdf/member/2026-07-10-c19c1c63-fbee-42db-a30c-edb712a66295.pdf.

[18] Ge E., Su M., Zhao R., Huang Z., Shan Y., Wei X. (2021). Geographical disparities in access to hospital care in ontario, canada: A spatial coverage modelling approach. BMJ Open.

[19] Heykoop C., Smrke A., Wolfe J., Rogers L.K., Hill T.T., Avery J., Okonkwo-Dappa A.J., Gill P.K., Weller S., Peacock S. (2025). Reshaping adolescent and young adult cancer care and support through participatory engagement. Int. J. Qual. Methods.

[20] Srikanthan A., Nagaratnam K., Das S., Siddiqui Z., Cleyn S., Surujballi J. (2025). Perspectives of Canadian adolescent and young adult (AYA) cancer survivors in designing a regional AYA survivorship program. J. Cancer Surviv..

[21] Tong A., Sainsbury P., Craig J. (2007). Consolidated criteria for reporting qualitative research (COREQ): A 32-item checklist for interviews and focus groups. Int. J. Qual. Health Care.

[22] Staniszewska S., Brett J., Simera I., Seers K., Mockford C., Goodlad S., Altman D.G., Moher D., Barber R., Denegri S. (2017). GRIPP2 reporting checklists: Tools to improve reporting of patient and public involvement in research. BMJ.

[23] Glaser B.G., Strauss A.L. (1967). The Discovery of Grounded Theory: Strategies for Qualitative Research.

[24] Braun V., Clarke V. (2006). Using thematic analysis in psychology. Qual. Res. Psychol..

[25] Kullman S.M., Chudyk A.M. (2025). Participatory member checking: A novel approach for engaging participants in co-creating qualitative findings. Int. J. Qual. Methods.

[26] Berkman A.M., Mittal N., Roth M.E. (2023). Adolescent and young adult cancers: Unmet needs and closing the gaps. Curr. Opin. Pediatr..

[27] Wong A.W.K., Chang T.T., Christopher K., Lau S.C.L., Beaupin L.K., Love B., Lipsey K.L., Feuerstein M. (2017). Patterns of unmet needs in adolescent and young adult (AYA) cancer survivors: In their own words. J. Cancer Surviv..

[28] Moura M.J., Dos Santos M.C., Barros L. (2025). Identifying concerns and needs in AYA survivors of pediatric cancer: A scoping review. Front. Psychol..

[29] Avutu V., Lynch K.A., Barnett M.E., Vera J.A., Glade Bender J.L., Tap W.D., Atkinson T.M. (2022). Psychosocial needs and preferences for care among adolescent and young adult cancer patients (ages 15–39): A qualitative study. Cancers.

[30] Tutelman P.R., Thurston C., Ranger T., Rader T., Henry B., Abdelaal M., Blue M., Buckland T.W., Del Gobbo S., Dobson L. (2026). Top 10 research priorities for adolescent and young adult cancer in Canada: A James Lind Alliance priority setting partnership. BMJ Open.

[31] Stout N.L., Fu J.B., Silver J.K. (2021). Prehabilitation is the gateway to better functional outcomes for individuals with cancer. J. Cancer Rehabil..

[32] Grimmett C., Heneka N., Chambers S. (2022). Psychological interventions prior to cancer surgery: A review of reviews. Curr. Anesthesiol. Rep..

[33] Psihogios A.M., Schwartz L.A., Deatrick J.A., Ver Hoeve E.S., Anderson L.M., Wartman E.C., Szalda D. (2019). Preferences for cancer survivorship care among adolescents and young adults who experienced healthcare transitions and their parents. J. Cancer Surviv..

[34] Lea S., Martins A., Bassett M., Cable M., Doig G., Fern L.A., Morgan S., Soanes L., Smith S., Whelan M. (2018). Issues experienced and support provided to adolescents and young adults at the end of active treatment for cancer: A rapid review of the literature. Eur. J. Cancer Care.

[35] Baudry V., Girodet M., Lochmann M., Bottichio M., Charton E., Flahault C., Baudry A.S., Bertrand A., Christophe V. (2024). Supportive care needs of adolescents and young adults 5 years after cancer: A qualitative study. Front. Psychol..

[36] Gebauer J., Baust K., Bardi E., Grabow D., Stein A., van der Pal H.J., Calaminus G., Langer T. (2020). Guidelines for long-term follow-up after childhood cancer: Practical implications for the daily work. Oncol. Res. Treat..

[37] Berkman A.M., Betts A.C., Beauchemin M., Parsons S.K., Freyer D.R., Roth M.E. (2024). Survivorship after adolescent and young adult cancer: Models of care, disparities, and opportunities. J. Natl. Cancer Inst..

[38] Shay L.A., Parsons H.M., Vernon S.W. (2017). Survivorship care planning and unmet information and service needs among adolescent and young adult cancer survivors. J. Adolesc. Young Adult Oncol..

[39] Lehmann V., Laan E.T.M., den Oudsten B.L. (2022). Sexual health-related care needs among young adult cancer patients and survivors: A systematic literature review. J. Cancer Surviv..

[40] Oveisi N., Cheng V., Taylor D., Kang P., Brotto L.A., Peacock S., McTaggart-Cowan H., Hanley G.E., Gill S., Rayar M. (2025). “Cancer changes everything, but it makes you wonder-Am I still enough?” Serial focus groups with adolescent and young adult cancer patients to understand experiences with cancer and sexual and reproductive health. BMC Cancer.

[41] Bhatia S., Pappo A.S., Acquazzino M., Allen-Rhoades W.A., Barnett M., Borinstein S.C., Casey R., Choo S., Chugh R., Dinner S. (2023). Adolescent and young adult (AYA) oncology, version 2.2024, NCCN clinical practice guidelines in oncology. J. Natl. Compr. Cancer Netw..

[42] Su H.I., Lacchetti C., Letourneau J., Partridge A.H., Qamar R., Quinn G.P., Reinecke J., Smith J.F., Tesch M., Wallace W.H. (2025). Fertility preservation in people with cancer: ASCO guideline update. J. Clin. Oncol..

[43] Government of Ontario Get Fertility Treatments. https://www.ontario.ca/page/get-fertility-treatments.

[44] Frederick N.N., Campbell K., Kenney L.B., Moss K., Speckhart A., Bober S.L. (2018). Barriers and facilitators to sexual and reproductive health communication between pediatric oncology clinicians and adolescent and young adult patients: The clinician perspective. Pediatr. Blood Cancer.

[45] Dai Y., Cook O.Y., Yeganeh L., Huang C., Ding J., Johnson C.E. (2020). Patient-reported barriers and facilitators to seeking and accessing support in gynecologic and breast cancer survivors with sexual problems: A systematic review of qualitative and quantitative studies. J. Sex. Med..

[46] Vadaparampil S., Kelvin J., Murphy D., Bowman M.L., Sehovic I., Quinn G. (2016). Fertility and fertility preservation: Scripts to support oncology nurses in discussions with adolescent and young adult patients. J. Clin. Outcomes Manag..

[47] Aubin S., Perez S. (2015). The clinician’s toolbox: Assessing the sexual impacts of cancer on adolescents and young adults with cancer (AYAC). Sex. Med..

[48] Waters A.R., van Thiel Berghuijs K.M., Kaddas H.K., Vaca Lopez P.L., Chevrier A., Ray N., Tsukamoto T., Allen K., Fair D.B., Lewis M.A. (2023). Sources of informal financial support among adolescent and young adult cancer survivors: A mixed methods analysis from the HIAYA CHAT study. Support. Care Cancer.

[49] McLoone J.K., Sansom-Daly U.M., Paglia A., Chia J., Larsen H.B., Fern L.A., Cohn R.J., Signorelli C. (2023). A scoping review exploring access to survivorship care for childhood, adolescent, and young adult cancer survivors: How can we optimize care pathways?. Adolesc. Health Med. Ther..

[50] Wood T.F., Murphy R.A. (2024). Tackling financial toxicity related to cancer care in Canada. Can. Med. Assoc. J..

[51] Smith G.L., Banegas M.P., Acquati C., Chang S., Chino F., Conti R.M., Greenup R.A., Kroll J.L., Liang M.I., Pisu M. (2022). Navigating financial toxicity in patients with cancer: A multidisciplinary management approach. CA Cancer J. Clin..

[52] Ehrhardt M.J., Friedman D.N., Hudson M.M. (2024). Health care transitions among adolescents and young adults with cancer. J. Clin. Oncol..

[53] Lorig K.R., Holman H. (2003). Self-management education: History, definition, outcomes, and mechanisms. Ann. Behav. Med..

[54] Pulvirenti M., McMillan J., Lawn S. (2014). Empowerment, patient centred care and self-management. Health Expect..

[55] Hardman R., Begg S., Spelten E. (2020). What impact do chronic disease self-management support interventions have on health inequity gaps related to socioeconomic status: A systematic review. BMC Health Serv. Res..

[56] Pallin N.D., McHugh S.M., Carvalho M., Hegarty J., Connolly R.M., Browne J.P. (2024). Enablers and barriers to accessing self-management support services for those living with and beyond cancer: A qualitative study using the theoretical domains framework. Psycho-Oncology.

[57] Kirk J., Jespersen J., McKillop S. (2022). Early psychosocial contact for adolescents and young adults (AYAs) with cancer: The impact of the AYA oncology navigator. J. Adolesc. Young Adult Oncol..

[58] Fox R.S., Fowler B., Carrera J.B., Reichek J., Sanford S.D. (2022). Increasing access to psychosocial care for adolescents and young adults with cancer by integrating targeted navigation services. Psycho-Oncology.

[59] San Martin-Feeney D., Samborn S., Allemang B., Patton M., Punjwani Z., Pfister K., Ryan L., Guilcher G.M.T., Hamiwka L., Klarenbach S. (2025). Transition experiences of adolescents and young adults working with a patient navigator. Health Care Transit..

[60] Tay J., Jarrar M., Yeung T., Kichler J.C. (2026). Perspectives of adolescents and young adults, caregivers, and health care providers on regional cancer care: Qualitative study. JMIR Cancer.

[61] Abdel-Rahman O., McFarlane E., Ohm H., Chan A., Loewen S., Litalien A., Steed H., Walker J. (2025). Advances in cancer therapy require urgent changes to the oncology workforce. Can. Med. Assoc. J..

[62] Canadian Association of Provincial Cancer Agencies (2025). Pan-Canadian Strategic Oncology Workforce Report: A Focus on Key Oncology Professions. https://capca.ca/wp-content/uploads/2025/05/CAPCA-Strategic-Oncology-Workforce-FINAL-2025-05-05.pdf.

[63] Signorelli C., Hoeg B.L., Asuzu C., Centeno I., Estape T., Fisher P., Lam W., Levkovich I., Manne S., Miles A. (2024). International survey of psychosocial care for cancer survivors in low-/middle- and high-income countries: Current practices, barriers, and facilitators to care. JCO Glob. Oncol..

[64] Ontario Health (Cancer Care Ontario) (2023). Optimizing Ambulatory Systemic Treatment Models of Care: Recommendation Report.

