# Supplementary material for: Exploring Key Unmet Supportive Care Needs of Adolescent and Young Adult Cancer Patients: A Qualitative Study to Inform Regional Program Development
Source: Curr Oncol. 2026 Jul 10;33(7):412. doi: 10.3390/curroncol33070412 (PMC13408510; doi:10.3390/curroncol33070412)
Supplement: Supplementary file 1 [file curroncol-33-00412-s001.zip › Curr Oncol_Supplementary File S1.pdf]

Supplementary File S1:
Consolidated Criteria for Reporting Qualitative Research (COREQ) Checklist

This file reports on qualitative research methods and reporting using the COREQ checklist. This table summarizes were relevant information about the research team and reflexivity, study design, and analysis and findings can be found in the manuscript. Items not applicable to the present study are noted as “N/A.”

Item #	Item	Description	Manuscript Section
**Domain 1: Research Team and Reflexivity**			
Personal Characteristics			
1	Interviewer/ Facilitator	Which author/s conducted the interview or focus group?	Interview
2	Credentials	What were the researcher’s credentials? E.g. PhD, MD	Interview
3	Occupation	What was their occupation at the time of the study?	Interview
4	Gender	Was the researcher male or female?	Interview
5	Experience and Training	What experience or training did the researcher have?	Interview
Relationship with Participants			
6	Relationship Established	Was a relationship established prior to study commencement?	N/A
7	Participant Knowledge of the Interviewer	What did the participants know about the researcher (e.g., personal goals, reasons for doing the research)?	N/A
8	Interviewer Characteristics	What characteristics were reported about the inter viewer/facilitator (e.g., bias, assumptions, reasons and interests in the research topic)?	N/A
**Domain 2: Study Design**			
Theoretical Framework			
9	Methodological orientation and theory	What methodological orientation was stated to underpin the study (e.g., grounded theory, discourse analysis, ethnography, phenomenology, content analysis)?	Study Design
Participant Selection			
10	Sampling	How were participants selected (e.g., purposive, convenience, consecutive, snowball)?	Participants and Procedure
11	Method of Approach	How were participants approached (e.g., face-to-face, telephone, mail, email)?	Participants and Procedure
12	Sample Size	How many participants were in the study?	Participants and Procedure
13	Non-Participation	How many people refused to participate or dropped out? What were the reasons for this?	N/A
Setting			
14	Setting of Data Collection	Where was the data collected (e.g., home, clinic, workplace)?	Interview
15	Presence of Non-Participants	Was anyone else present besides the participants and researchers?	Interview
16	Description of Sample	What are the important characteristics of the sample (e.g., demographic data, date)?	Sample
Data Collection			
17	Interview Guide	Were questions, prompts, guides provided by the authors? Was it pilot tested?	Interview; Table 1; Additional File 3
18	Repeat Interviews	Were repeat interviews carried out? If yes, how many?	N/A

19	Audio/Visual Recording	Did the research use audio or visual recording to collect the data?	Interview
20	Field Notes	Were field notes made during and/or after the interview or focus group?	Interview
21	Duration	What was the duration of the interviews or focus group?	Interview
22	Data Saturation	Was data saturation discussed?	Sample Size
23	Transcripts Returned	Were transcripts returned to participants for comment and/or correction?	Data Analysis
**Domain 3: Analysis and Findings**			
Data Analysis			
24	Number of Data Coders	How many data coders coded the data?	Data Analysis
25	Description of the Coding Tree	Did authors provide a description of the coding tree?	N/A
26	Derivation of Themes	Were themes identified in advance or derived from the data?	Data Analysis
27	Software	What software, if applicable, was used to manage the data?	Data Analysis
28	Participant Checking	Did participants provide feedback on the findings?	Data Analysis; Additional File 2
Reporting			
29	Quotations Presented	Were participant quotations presented to illustrate the themes/findings? Was each quotation identified (e.g., participant number)?	Themes
30	Data and Findings Consistent	Was there consistency between the data presented and the findings?	Themes; Discussion
31	Clarity of Major Themes	Were major themes clearly presented in the findings?	Themes
32	Clarity of Minor Themes	Is there a description of diverse cases or discussion of minor themes?	Themes

Developed from: Tong, A., Sainsbury, P., & Craig, J. (2007). Consolidated criteria for reporting qualitative research (COREQ): A 32-item checklist for interviews and focus groups. *International Journal for Quality in Health Care, 19*(6), 349–357. https://doi.org/10.1093/intqhc/mzm042
