# Supplementary material for: Exploring Key Unmet Supportive Care Needs of Adolescent and Young Adult Cancer Patients: A Qualitative Study to Inform Regional Program Development
Source: Curr Oncol. 2026 Jul 10;33(7):412. doi: 10.3390/curroncol33070412 (PMC13408510; doi:10.3390/curroncol33070412)
Supplement: Supplementary file 1 [file curroncol-33-00412-s001.zip › Curr Oncol_Supplementary File S2.pdf]

Supplementary File S2:
Guidance for Reporting Involvement of Patients and the Public (GRIPP2) Checklist

This file reports on patient partner involvement using the GRIPP2 short-form checklist. This table summarizes how patient partners contributed to the study and identifies where relevant information about patient and public involvement can be found in the manuscript.

Section and Topic	Item	Manuscript Section
**Aim**	*Report the aim of PPI in the study.*<br><br>Patient partners were involved to support development of a regional AYA cancer program that reflected the needs, priorities, and experiences of patients at the TOH *Champlain Regional Cancer Program*. In this study, PPI aimed to enhance the relevance and patient-centredness of the data collection tools, interpretation of findings, and resulting program development.	Background; Study Design
**Methods**	*Provide a clear description of the methods used for PPI in the study.*<br><br>Patient partners played a key role in the development and refinement of the semi-structured interview guide. Specifically, they helped shape interview questions and reviewed them to ensure clarity, relevance, sensitivity, neutrality, and feasibility. Further, one patient partner volunteered to pilot-test the guide before interviews were conducted.	Interview
**Study Results**	*Outcomes—Report the results of PPI in the study, including both positive and negative outcomes.*<br><br>PPI contributed to the study in the following ways:<br>• Patient partners helped to develop, refine, and pilot-test the interview tool.<br>• Patient partners reviewed preliminary interview findings with the research team to guide interpretation and the initial launch of the regional program.<br>• Patient partners critically reviewed the manuscript and offered additional insights that were incorporated throughout the write-up.<br>• Overall PPI helped ensure that data collection and interpretation were grounded in patient-identified priorities and core supportive care needs.	Interview; Declarations
**Discussion and Conclusions**	*Comment on the extent to which PPI influenced the study overall. Describe positive and negative effects.*<br><br>PPI enhanced the study's patient-centredness by helping ensure that data collection focused on issues relevant to AYAs' unmet supportive care needs. Patient partners' contributions also supported interpretation of the findings by helping the research team identify which results were most meaningful from a patient perspective and how these insights could inform the development of a regional AYA cancer program.	Discussion; Declarations
**Reflections**	*Critical perspective—Comment critically on the study, reflecting on the things that went well and those that did not, so others can learn from this experience.*<br><br>PPI strengthened the relevance of the study and will be instrumental in developing the regional cancer program. However, despite efforts to achieve sample variation, the perspectives represented may not reflect the full diversity of the broader TOH *Champlain Regional Cancer Program* population. This may	Limitations and Strengths

	reflect interviewer availability and study language constraints, as well as barriers faced by non-respondents (e.g., caregiving responsibilities, scheduling constraints, lack of childcare).	

Notes. PPI=patient and public involvement; TOH=*The Ottawa Hospital*.

Developed from: Staniszewska, S., Brett, J., Simera, I., Seers, K., Mockford, C., Goodlad, S., Altman, D.G., Moher, D., Barber, R., Denegri, S., Entwistle, A., Littlejohns, P., Morris, C., Suleman, R., Thomas, V., & Tysall, C. (2017). GRIPP2 reporting checklists: Tools to improve reporting of patient and public involvement in research. *BMJ*, *358*, j3453. https://doi.org/10.1136/bmj.j3453
