# Supplementary material for: Exploring Key Unmet Supportive Care Needs of Adolescent and Young Adult Cancer Patients: A Qualitative Study to Inform Regional Program Development
Source: Curr Oncol. 2026 Jul 10;33(7):412. doi: 10.3390/curroncol33070412 (PMC13408510; doi:10.3390/curroncol33070412)
Supplement: Supplementary file 1 [file curroncol-33-00412-s001.zip › Curr Oncol_Supplementary File S3.pdf]

Supplementary File S3: Interview Guide

Introduction

Thank you for meeting with us today. We are completing this interview to learn about your experiences with cancer so we can develop a regional program to provide better care for adolescents and young adults with cancer. This interview will be kept confidential. No one else will know that you participated, including your care providers. We will be recording the interview so that we can transcribe the interviews. Anything that you or I say that could identify you will be removed from the transcription. Also, the recording will be deleted after transcription. Please let us know if you have any questions or concerns at any times.

The questions we ask may bring up challenging feelings and memories. You can take a break at any point. You can choose not to answer questions. If you'd like to skip a question, you can say "skip" and we will move on without asking you why you want to skip the question. If you would like to speak with a mental health professional after the interview, we will connect you with one.

Section 1: Cancer Diagnosis

Understanding the Diagnosis

- Can you tell me about when and how you were diagnosed?
    - Did it take a long time to receive your diagnosis?
- How did you share the news with your family and friends?
- What was the most challenging part of receiving your diagnosis?
- Has your understanding of cancer changed since your diagnosis, and if so, how?

Impact on Daily Life

- How did your daily life change after your diagnosis?
    - Are there still changes to your daily life?
- What activities or hobbies bring you joy and help you feel like yourself?
- What were the most significant adjustments you had to make?

Section 2: Mental Health

Emotional Wellbeing

- How have you been feeling emotionally since your diagnosis?
    - Have your feelings changed over time?
- What coping mechanisms have you found to be most helpful?
- Are there specific challenges or circumstances that affect your mental health?

Support Systems

- Who do you turn to for emotional support?
- Have you looked for professional mental health support? If so, how has it helped you?
- How do you see your life after treatment? If in survivorship, how has your life changed since treatment?

- Do you have hopes and goals for the future?
    - How have these changed since diagnosis?

Section 3: Fertility

Fertility Concerns

The following questions are about fertility, or your ability to have biological children. We will also ask about fertility preservation, which can include egg, sperm, and/or embryo freezing.

- Were you told about the potential impact of your cancer treatments on your fertility/ability to have kids?
- What information or resources did you receive about fertility preservation?
- Have you considered or undergone any fertility preservation methods? When did you undertake this and what were they?

Future Family Planning

- How do you feel about the possibility of starting a family in the future?
- What are your biggest concerns regarding fertility and family planning?
- What kind of support would you like to receive on this topic?

Section 4: Sexuality

Changes in Sexual Health

- How has your diagnosis and treatment affected your sexual health?
- What challenges have you faced in sexual activity?
- Have you noticed any changes in your body image/how you see yourself and your body or self-esteem?

Support and Resources

- What kind of information or resources have you received about sexual health?
- Are there any specific topics related to sexuality that you would like more information about?
- How comfortable do you feel discussing sexual health with your healthcare team?

Conclusion

To wrap up, we would like to ask some demographic questions to make sure we interview patients from a variety of backgrounds.

1 - Age at diagnosis:
2 - Current age:
3- Relationship Status at diagnosis:
4 - Current relationship status:
5 - Number of children at diagnosis:
6 - Employment status at diagnosis:
7 - Highest education status attained:
8 - Current employment status:

9 - Type of cancer and stage at diagnosis:
10 - Current stage:
11 - First three digits of postal code:
If comfortable disclosing:
12 - Race
13 - Religions
14 - Gender identity

Is there anything else you'd like to discuss or think would be helpful for us to know about teens/young adults' experience with cancer? Do you have any questions for us?

Thank you for sharing your experiences and insights.
